# Design of simple nonovershooting controllers for linear high order systems with or without time delay

**DOI:** 10.1038/s41598-023-49802-x

**Published:** 2024-01-09

**Authors:** Huanchao Du, Bobo Feng, Jieshi Shen, Dan Li

**Affiliations:** 1 Xi’an University of Posts and Telecommunications, Shaanxi, China; 2 Chinese Aeronautical Radio and Electronics Research Institute, Shanghai, China; 3 China Electronic Product Reliability and Environmental Testing Research Institute, Guangzhou, China; 4 Guangdong Provincial Key Laboratory of Electronic Information Products Reliability Technology, Guangzhou, China

**Keywords:** Engineering, Electrical and electronic engineering, Mechanical engineering

## Abstract

In this paper, we mainly considered the problem of nonovershooting control of high order systems with or without time delay by simple controllers. As basic principles for nonovershooting control systems, three propositions are offered and proved. Under direction of these principles, a nonovershooting dominant pole control structure having three dominant poles, i.e., one real pole and a pair of complex conjugate poles on its left, is proposed. While its zeroes and nondominant poles are on the left side of these three dominant poles with sufficient distance. The controllers adopted are composed by first order filter and PD-PID controller. Dominance of the three dominant poles can be checked and ensured through the computational method we offered. Two illustrating examples are given to show the effectiveness of our method.

## Introduction

Nonovershooting controllers has drawn the attention of control engineers for decades due to their wide range of applications, such as filter design in circuits^1^, high precision position control in lithographic equipment^2,3^ and vibration reduction control in aircrafts and vehicles^4,5^. Usually, the plants to be controlled are of high order as their mathematical models becoming more complete and multi-input multi-output form are considered. When time delay is included, the plants’ model will become even more complex. Methods for determining whether given systems having nonovershooting step responses have been offered in Du^6–8^, however, their methods are based on sufficient conditions and can be applied to certain systems such as systems having only real poles. Despite the recent development in nonovershooting control^8–11^, available necessary and sufficient conditions for systems having nonovershooting step responses are still unfound.

To avoid the above problem, a common practice for nonovershooting control is to place all the closed loop poles on the negative real axis by arbitrary pole placement technique^10^, and reader can refer to Shumafov^12^ for a through survey of the pole placement control of linear systems. The disadvantage of this method is that the order of the designed systems will be very high, since arbitrary pole placement requires high order controllers be used for higher order plants^13^. It is pointed out by Da-Wei Gu et al. that high order controllers will lead to cost, commissioning, reliability, maintenance problems during implementation and are not welcomed by practicing control engineers^14^. Restricted by the simple structure, only few poles can be placed to designated location by simple controllers such as PID controllers^13,15–17^, and simple nonovershooting control is hard to realize. Besides, the arbitrary pole placement technique will fail as infinite number of poles will be introduced by the existence of time delay.

As a compromise, dominant pole placement method by PID controllers becomes the most implementable one for controlling of high order systems^13^. In the work of Ma et al.^16,18^, achievable delay margin of low order systems is studied, and explicit bounds for stabilization of second order unstable delay systems are offered by PID controllers. Boussaada et al.^19^ discussed the recent results on maximal multiplicity induced dominancy for spectral values in reduced order time delay systems, and they extend these results to a general class of second order retarded differential equations. Pekař et al.^20^ analyzed the pole loci and characteristic function of simple delayed model, and applied the infinite-dimensional model to a complex heating–cooling process with heat exchangers. For systems with delays, Pekař and Matušů^21^ offered a suboptimal shifting based pole-zero placement method which is essentially also a dominant pole placement method. Dimensional analysis approach raised by Zítek et al.^17^ can place three dominant poles in delayed PID control loops. And Das et al.^22^ convert the delays into finite number of poles for dominant pole placement by PID controllers.

When referring to nonovershooting control, to the best of the author’s knowledge, no method is available to guarantee nonovershooting control of systems having time delay, not to mention by using simple PID controllers. Thus, it is rather desirable to find ways of designing simple nonovershooting controllers for high order systems with or without time delay. The main idea of our method is that: By using simple controllers such as first order low-pass filter and PID controllers, systems with three dominant poles, i.e., one real pole and a pair of complex conjugate poles, can be designed according to dominant pole placement method. The dominant real pole is supplied by either PID controller or first order low-pass filter. If this real pole is supplied by PID controller, structure of the controller can be further simplified with first order low-pass filter omitted. Nonovershooting step responses can be realized through proper placement of these three dominant poles.

**Figure 1:**
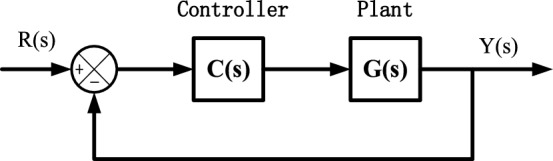
Control structure.

In this paper, linear time invariant (LTI) systems are considered, and the paper is organized as follows: In “Basic principles for nonovershooting” Section basic principles for systems having nonovershooting step responses are discussed. Control structure is stated in “Dominant pole control structure” Section, and then pole placement method is presented in “Dominant pole placement” Section. Numerical examples and conclusions are given in “Illustrating examples” and “Conclusions” Sections respectively. The control structure considered in this paper is given in Fig. 1.

## Basic principles for nonovershooting

Here, discussed are three basic principles that the poles and zeros of the nonovershooting systems should follow. For convenience, all transfer functions are assumed to have a unit static gain in the following content.

### Proposition 1

A necessary condition for stable, rational, strictly proper transfer functions to have nonovershooting step responses is that the dominant poles of these transfer functions must be real.

### Proof

The stable, rational, strictly proper transfer functions are described as.

1 $$G\left( s \right) = K\frac{{s^{m} + b_{m - 1} s^{m - 1} + \cdots + b_{1} s + b_{0} }}{{s^{n} + a_{n - 1} s^{n - 1} + \cdots + a_{1} s + a_{0} }} = K\frac{{\left( {s + z_{1} } \right)\left( {s + z_{2} } \right) \cdots \left( {s + z_{m} } \right)}}{{\left( {s + p_{1} } \right)\left( {s + p_{2} } \right) \cdots \left( {s + p_{n} } \right)}}, m < n{ }$$

where $K$ is a scaling factor; $a_{0}$, …, $a_{n - 1}$ and $b_{0}$,…, $b_{m - 1}$ are coefficients of denominator and numerator polynomial of $G\left( s \right)$; $- z_{1}$,…, $- z_{m}$ and $- p_{1}$, …, $- p_{n}$ are its zeros and poles. For convenience, we would assume that $K = \frac{{a_{0} }}{{b_{0} }} = \frac{{p_{1} p_{2} \cdots p_{n} }}{{z_{1} z_{2} \cdots z_{m} }}$, which means that $G\left( s \right)$ has a unit static gain. Among all poles, dominant pole is the one having the largest real parts, it may be a real pole or a pair of complex conjugate poles, these dominant poles are also called leading poles in Zítek^17^.

Define $$\overline{G}\left( s \right) = 1 - G\left( s \right),$$ then

2 $$\overline{G}\left( s \right) = \frac{sN\left( s \right)}{{s^{n} + a_{n - 1} s^{n - 1} + \cdots + a_{1} s + a_{0} }}$$

with

$$N\left( s \right) = s^{n - 1} + a_{n - 1} s^{n - 2} + \cdots + a_{2} s + a_{1} - \frac{{a_{0} }}{{b_{0} }}\left( {s^{m - 1} + b_{m - 1} s^{m - 2} + \cdots + b_{2} s + b_{1} } \right)$$

**Figure 2:**
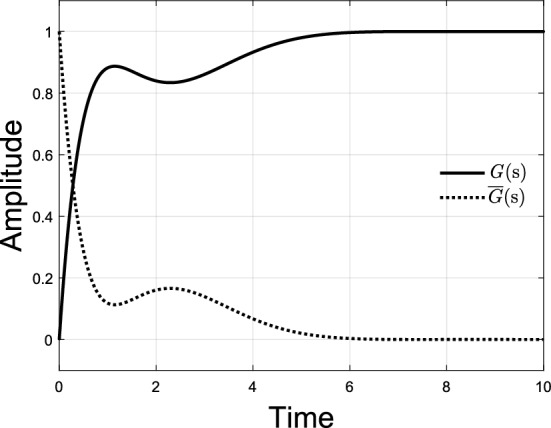
Unit step responses of $G\left( s \right)$ and $\overline{G}\left( s \right)$.

If $G\left( s \right)$ has a nonovershooting step response as shown in Fig. 2, then $\overline{G}\left( s \right)$ would have a nonnegative step response also shown in Fig. 2. Note that the step response of $\overline{G}\left( s \right)$ is also the impulse response of $\overline{G}\left( s \right)/s$. By introducing $\overline{G}\left( s \right)$, the concept that $G\left( s \right)$ has a nonovershooting step response is equivalent to that $\overline{G}\left( s \right)/s$ has a nonnegative impulse response. Moreover, $G\left( s \right)$ and $\overline{G}\left( s \right)/s$ have the same poles.

Assume that $\overline{G}\left( s \right)/s$ has a nonnegative impulse response, and its dominant poles are several pair of complex conjugate poles with the same real part and independent multiplicity. Denotes these dominant complex conjugate poles as $- \alpha \pm j\beta_{i}$, $i = 1, \cdots ,l$, with multiplicity of $\kappa_{i}$ each. Equation (2) can be rewritten as

3 $$\frac{1}{s}\overline{G}\left( s \right) = \frac{N\left( s \right)}{{\left( {\left( {s + \alpha } \right)^{2} + \beta_{1}^{2} } \right)^{{\kappa_{1} }} \cdots \left( {\left( {s + \alpha } \right)^{2} + \beta_{l}^{2} } \right)^{{\kappa_{l} }} D_{1} \left( s \right)}}$$

with $D_{1} \left( s \right) = \mathop \prod \limits_{d = 1}^{{\left( {n - 2\mathop \sum \limits_{i = 1}^{l} \kappa_{i} } \right)}} \left( {s + p_{d} } \right)$, and $\left\{ {\left( {{\text{Real part of }}\left( { - p_{d} } \right)} \right) < - \alpha {}|_{{d = 1, \cdots ,\left( {n - 2\mathop \sum \limits_{i = 1}^{l} \kappa_{i} } \right)}} } \right\}.$ By inverse Laplace transform, the time domain impulse response of $\overline{G}\left( s \right)/s$ can be written as

4 $$g_{\Delta } \left( t \right) = e^{ - \alpha t} \mathop \sum \limits_{i = 1}^{l} \left[ {\varphi_{i} \left( t \right)\cos \left( {\beta_{i} t} \right) + \phi_{i} \left( t \right)\sin \left( {\beta_{i} t} \right)} \right] + e^{{ - \left( {\alpha + \varepsilon } \right)t}} \delta \left( t \right)$$

with

$$\varphi _{i} \left( t \right) = \varphi _{{i_{{\left( {\kappa _{i} - 1} \right)}} }} \cdot t^{{\kappa _{i} - 1}} + \cdots + \varphi _{{i_{1} }} \cdot t + \varphi _{{i_{0} }}$$

$$\phi _{i} \left( t \right) = \phi _{{i_{{\left( {\kappa _{i} - 1} \right)}} }} \cdot t^{{\kappa _{i} - 1}} + \cdots + \phi _{{i_{1} }} \cdot t + \phi _{{i_{0} }}$$

where $\left\{ {\varphi_{{i_{k} }} ,\phi_{{i_{k} }} |_{{k = 0,1 \cdots ,\kappa_{i} - 1}} } \right\}$ are constant coefficients, $e^{{ - \left( {\alpha + \varepsilon } \right)t}} \delta \left( t \right)$ are response items corresponding to the nondominant left half plane (LHP) poles with $\left\{ { - \left( {\alpha + \varepsilon } \right) = {\text{max}}\left( {{\text{Real part of }}\left( { - p_{d} } \right){}|_{{d = 1, \cdots ,\left( {n - 2\mathop \sum \limits_{i = 1}^{l} \kappa_{i} } \right)}} } \right)} \right\}$ and $\varepsilon > 0$. Suppose $\kappa_{{{\text{max}}}} = {\text{max}}\left( {\kappa_{i} |_{i = 1, \cdots ,l} } \right)$, when $t$ approaches infinity, the following approximation can be obtained,

5 $$\begin{aligned} \mathop {\lim }\limits_{t \to + \infty } g_{\Delta } \left( t \right) = & e^{ - \alpha t} \left\{ {\mathop \sum \limits_{i = 1}^{l} \left[ {\varphi_{i} \left( t \right)\cos \left( {\beta_{i} t} \right) + \phi_{i} \left( t \right)\sin \left( {\beta_{i} t} \right)} \right] + e^{ - \varepsilon t} \delta \left( t \right)} \right\} \\ \approx & e^{ - \alpha t} t^{{\left( {\kappa_{{{\text{max}}}} - 1} \right)}} \mathop \sum \limits_{{i = 1, \kappa_{i} = \kappa_{{{\text{max}}}} }}^{l} \left[ {\varphi_{{i_{{\left( {\kappa_{i} - 1} \right)}} }} \cos \left( {\beta_{i} t} \right) + \phi_{{i_{{\left( {\kappa_{i} - 1} \right)}} }} \sin \left( {\beta_{i} t} \right)} \right] \\ \approx & e^{ - \alpha t} t^{{\left( {\kappa_{{{\text{max}}}} - 1} \right)}} T\left( t \right) \\ \end{aligned}$$

with $T\left( t \right)$ defined as follows,

6 $$T\left( t \right) = \mathop \sum \limits_{{i = 1,\kappa_{i} = \kappa_{{{\text{max}}}} }}^{l} A_{i} \sin \left( {\beta_{i} t + \theta_{i} } \right)$$

$$A_{i} = \sqrt {\left( {\varphi_{{i_{{\left( {\kappa_{i} - 1} \right)}} }} } \right)^{2} + \left( {\phi_{{i_{{\left( {\kappa_{i} - 1} \right)}} }} } \right)^{2} }$$

$$\theta_{i} = \arcsin \left( {\frac{{\varphi_{{i_{{\left( {\kappa_{i} - 1} \right)}} }} }}{{A_{i} }}} \right)$$

Addition of 2 arbitrary sine functions $A_{1} \sin \left( {\beta_{1} t + \theta_{1} } \right)$ and $A_{2} \sin \left( {\beta_{2} t + \theta_{2} } \right)$ can be changed into the following form,

7 $$A_{1} \sin \left( {\beta_{1} t + \theta_{1} } \right) + A_{2} \sin \left( {\beta_{2} t + \theta_{2} } \right) = A\sin \left( {\frac{{\beta_{1} + \beta_{2} }}{2}t + \frac{{\theta_{1} + \theta_{2} }}{2} + \psi } \right)$$

where

$$A = \sqrt {A_{1}^{2} + A_{2}^{2} + A_{1} A_{2} {\text{cos}}\left( {\left( {\beta_{1} - \beta_{2} } \right)t + \left( {\theta_{1} - \theta_{2} } \right)} \right)}$$

$$\psi = {\text{arctan}}\left( {\frac{{A_{1} - A_{2} }}{{A_{1} + A_{2} }}{\text{tan}}\left( {\frac{{\beta_{1} - \beta_{2} }}{2}t + \frac{{\theta_{1} - \theta_{2} }}{2}} \right)} \right)$$

According to Eq. (7), $T\left( t \right)$ of Eq. (6) can be rewritten as

8 $$T\left( t \right) = {\varvec{A}}\left( {A_{1} , \cdots A_{l} ,\beta_{1} , \cdots ,\beta_{l} ,\theta_{1} , \cdots ,\theta_{l} } \right)\sin \left( {{{\varvec{\Omega}}}\left( {\beta_{1} , \cdots ,\beta_{l} } \right)t + {{\varvec{\Theta}}}\left( {A_{1} , \cdots A_{l} ,\beta_{1} , \cdots ,\beta_{l} ,\theta_{1} , \cdots ,\theta_{l} } \right)} \right)$$

where ${\varvec{A}}\left( \cdot \right)$, ${{\varvec{\Omega}}}\left( \cdot \right)$ and ${{\varvec{\Theta}}}\left( \cdot \right)$ are functions for amplitude, radian frequency and phase shift in a broad sense, since both ${\varvec{A}}\left( \cdot \right)$ and ${{\varvec{\Theta}}}\left( \cdot \right)$ are time variant variables.

As $T\left( t \right)$ is a continuous sine function of time, it will change from positive to negative and then positive repeatedly when time goes on. According to Eq. (5), $g_{\Delta } \left( t \right)$ is bound to have negative values repeatedly when time goes to infinity, which contradicts to the assumption that $g_{\Delta } \left( t \right)$ has a nonnegative impulse response. Therefore, $\overline{G}\left( s \right)/s$ would have an impulse with negative values, and $G\left( s \right)$ would have an overshooting step response.

Based on the above, it is impossible for transfer functions to have nonovershooting step responses when they have complex dominant poles.$\square$

For the control structure described by Fig. 1, the following results can be obtained.

### Proposition 2

If the closed-loop transfer function with only one integrator in the forward path and a unit feedback has a nonovershooting step response, both open loop real zeros and real poles with the pole at origin excluded must lie to the left of the closed-loop dominant real poles.

### Proof

Denote the open loop transfer function $G_{open} \left( s \right)$ as follows,

9 $$G_{{{\text{open}}}} \left( s \right) = \frac{N\left( s \right)}{{D\left( s \right)}} = \frac{{\left( {s + z_{1} } \right)\left( {s + z_{2} } \right) \cdots \left( {s + z_{m} } \right)}}{{s\left( {s + d_{1} } \right) \cdots \left( {s + d_{{n_{1} }} } \right)D_{s} \left( s \right)}}$$

where $- d_{i} |_{{i = 1, \cdots ,n_{1} }}$ are real poles, $D_{s} \left( s \right)$ is a polynomial composed only by complex conjugate poles; The poles of $G_{{{\text{open}}}} \left( s \right)$ can be LHP or right half plane (RHP) poles; $- z_{i} |_{i = 1, \cdots ,m}$ are zeros of $G_{{{\text{open}}}} \left( s \right)$.

Then the closed-loop transfer function can be written as

10 $$G_{{{\text{close}}}} \left( s \right) = \frac{N\left( s \right)}{{D\left( s \right) + N\left( s \right)}} = \frac{{\left( {s + z_{1} } \right)\left( {s + z_{2} } \right) \cdots \left( {s + z_{m} } \right)}}{{\left( {s + p_{1} } \right)\left( {s + p_{2} } \right) \cdots \left( {s + p_{n} } \right)}},\,\,m < n$$

where $- p_{i} |_{i = 1, \cdots ,n}$ are poles of $G_{{{\text{close}}}} \left( s \right)$.

Define $\overline{G}_{{{\text{close}}}} \left( s \right) = 1 - G_{{{\text{close}}}} \left( s \right)$, and it can be written as

11 $$\overline{G}_{{{\text{close}}}} \left( s \right) = s\overline{G}_{{{\text{open}}}} \left( s \right) = \frac{{s\left( {s + d_{1} } \right) \cdots \left( {s + d_{{n_{1} }} } \right)D_{s} \left( s \right)}}{{\left( {s + p_{1} } \right)\left( {s + p_{2} } \right) \cdots \left( {s + p_{n} } \right)}}$$

The following proving process can be divided into two steps:

*Step 1*. We would prove that when $G_{{{\text{close}}}} \left( s \right)$ has a nonovershooting step response, the open loop real poles with the pole at origin excluded must lie to the left of the closed-loop dominant real poles.

If $G_{{{\text{close}}}} \left( s \right)$ has a nonovershooting step response, i.e., $G_{{{\text{close}}}} \left( s \right)/s$, then $\overline{G}_{{{\text{close}}}} \left( s \right)$ would have a unit step response $\overline{G}_{{{\text{close}}}} \left( s \right)/s$ as follows,

12 $$\frac{1}{s}\overline{G}_{{{\text{close}}}} \left( s \right) = \overline{G}_{{{\text{open}}}} \left( s \right) = \frac{1}{s} - \frac{1}{s}G_{{{\text{close}}}} \left( s \right)$$

Similar to the relationship between unit step responses of $G\left( s \right)$ and $\overline{G}\left( s \right)$ in Fig. 2, we know that the unit step response of $\overline{G}_{close} \left( s \right)$ is nonnegative, or equivalently $\overline{G}_{{{\text{open}}}} \left( s \right)$ has a nonnegative impulse response. It is pointed out in Widder’s theorem^23^ that all the real zeros of $\overline{G}_{{{\text{open}}}} \left( s \right)$ with a nonnegative impulse response must lie to the left of its closed-loop dominant real poles. Since all zeros of $\overline{G}_{{{\text{open}}}} \left( s \right)$ are poles of $G_{{{\text{open}}}} \left( s \right)$, therefore, the open loop real poles with the pole at origin excluded must lie to the left of the closed-loop dominant real poles when corresponding closed-loop transfer function has a nonovershooting step response.

*Step 2*. We would prove that when $G_{{{\text{close}}}} \left( s \right)$ has a nonovershooting step response, the open loop real zeros must lie to the left of the closed-loop dominant real poles.

Suppose $G_{{{\text{close}}}} \left( s \right)$ has a nonovershooting step response with $- p_{1}$ be its dominant real pole, and $G_{{{\text{open}}}} \left( s \right)$ has at least one real zero lying to the right of $- p_{1}$, denote this zero as $- z_{1}$ with $p_{1} > z_{1} \ne 0$ holds. According to step 1, we know that all real poles of $G_{{{\text{open}}}} \left( s \right)$, with the pole at origin excluded, are on LHP when $G_{{{\text{close}}}} \left( s \right)$ has a nonovershooting step response. Therefore, $d_{i} > 0|_{{i = 1, \cdots ,n_{1} }}$ holds.

When $s = - z_{1}$, $D\left( { - z_{1} } \right) + N\left( { - z_{1} } \right) > 0$ holds according to above content and Eq. (8). As $N\left( { - z_{1} } \right) = 0$, $D\left( { - z_{1} } \right) > 0$ can be easily found.

Choose $s = - \varepsilon$ with $\varepsilon$ satisfying that $\left\{ {0 < \varepsilon < \min \left( {z_{1} ,d_{i} |_{{i = 1, \cdots ,n_{1} }} } \right)} \right\}$, $D\left( { - \varepsilon_{1} } \right) < 0$ holds since $\left( {s + d_{1} } \right) \cdots \left( {s + d_{{n_{1} }} } \right) > 0|_{{s = - \varepsilon_{1} }}$ and $D_{s} \left( { - \varepsilon_{1} } \right) > 0$. Determined by the polynomial roots’ theorem, we know that polynomial $D\left( s \right)$ must have a real root lying in $\left( { - z_{1} , - \varepsilon_{1} } \right)$, which means that $G_{{{\text{open}}}} \left( s \right)$ has a pole on the right of the closed-loop dominant pole $- p_{1}$. That contradicts to the result proved in step 1, therefore, when the closed-loop transfer function has a nonovershooting step response, the open loop real zeros must lie to the left of the closed-loop dominant real poles. $\square$

### Remarks 1.

It can be concluded from Proposition 2 that when the open loop transfer function has RHP real poles, overshoots must exist in the step response of closed-loop. And speed of the system’s nonovershooting response will be determined by the dominant open loop real pole.

The condition that open loop real zeros lie to the left of the closed-loop dominant real poles is a necessity not only for closed-loop transfer functions to have a nonovershooting step response, but also for closed-loop transfer functions to have a nondecreasing step response.

### Proposition 3

If $p > 0,\alpha > 0,\beta \ge 0,k > 0$, when $p \le \alpha$ and $r \ge \frac{1}{2}$, transfer functions $G_{1} \left( s \right) = \frac{k}{{\left( {s + p} \right)\left( {\left( {s + \alpha } \right)^{2} + \beta^{2} } \right)}}$ and $G_{2} \left( s \right) = \frac{{k\left( {\left( {s + \alpha } \right)^{2} + r\beta^{2} } \right)}}{{\left( {s + p} \right)\left( {\left( {s + \alpha } \right)^{2} + \beta^{2} } \right)}}$ would have nonovershooting step responses.

### Proof

*Step 1*. $G_{1} \left( s \right)$ can be rewritten as.

13 $$G_{1} \left( s \right) = G_{11} \left( s \right) + G_{12} \left( s \right)$$

where $$G_{11} \left( s \right) = \frac{k}{{\left( {s + \alpha } \right)\left( {\left( {s + \alpha } \right)^{2} + \beta^{2} } \right)}}$$ and $$G_{12} \left( s \right) = \frac{{\left( {\alpha - p} \right)}}{{\left( {s + p} \right)}}G_{11} \left( s \right)$$.

The impulse response of $G_{11} \left( s \right)$ is $y_{11} \left( t \right)$ as follows,

14 $$y_{11} \left( t \right) = \frac{k}{{\beta^{2} }}e^{ - \alpha t} \left( {1 - \cos \left( {\beta t} \right)} \right)$$

It is easy to find that $y_{11} \left( t \right) \ge 0$ for $\forall t \ge 0$, then $G_{11} \left( s \right)$ has a nonnegative impulse response. As $p \le a$, $\frac{{\left( {\alpha - p} \right)}}{{\left( {s + p} \right)}}$ also has a nonnegative impulse, i.e., $\left( {\alpha - p} \right)e^{ - pt} > 0$ for $\forall t \ge 0$. Impulse response of $G_{12} \left( s \right)$ is nonnegative since it is a convolution of two nonnegative impulse responses. Therefore, $G_{1} \left( s \right)$ has a nonnegative impulse response, i.e., $G_{1} \left( s \right)$ has a nonovershooting step response.

*Step 2*: Use the same method given in step one, $G_{2} \left( s \right)$ can be rewritten as

15 $$G_{2} \left( s \right) = G_{21} \left( s \right) + G_{22} \left( s \right)$$

where $$G_{21} \left( s \right) = \frac{{k\left( {\left( {s + \alpha } \right)^{2} + r\beta^{2} } \right)}}{{\left( {s + \alpha } \right)\left( {\left( {s + \alpha } \right)^{2} + \beta^{2} } \right)}}$$ and $$G_{22} \left( s \right) = \frac{{\left( {\alpha - p} \right)}}{{\left( {s + p} \right)}}G_{21} \left( s \right)$$.

Further, $G_{21} \left( s \right)$ can be rewritten as

16 $$G_{21} \left( s \right) = \frac{k}{2}\left( {\frac{1}{s + \alpha } + \frac{s + \alpha }{{\left( {s + \alpha } \right)^{2} + \beta^{2} }} + \frac{{\left( {2r - 1} \right)\beta^{2} }}{{\left( {s + \alpha } \right)\left( {\left( {s + \alpha } \right)^{2} + \beta^{2} } \right)}}} \right)=\frac{k}{2}\left( {G_{211} \left( s \right) + G_{212} \left( s \right)} \right)$$

where

$$G_{211} \left( s \right) = \frac{1}{s + \alpha } + \frac{s + \alpha }{{\left( {s + \alpha } \right)^{2} + \beta^{2} }}$$

$$G_{212} \left( s \right) = \frac{{\left( {2r - 1} \right)b^{2} }}{{\left( {s + \alpha } \right)\left( {\left( {s + \alpha } \right)^{2} + \beta^{2} } \right)}}$$

The impulse responses of $G_{21} \left( s \right)$, $G_{211} \left( s \right)$ and $G_{212} \left( s \right)$ are denoted as $y_{21} \left( t \right)$, $y_{211} \left( t \right)$ and $y_{212} \left( t \right)$ with

17 $$y_{21} \left( t \right) = \frac{k}{2}\left( {y_{211} \left( t \right) + y_{212} \left( t \right)} \right)$$

where

$$y_{211} \left( t \right) = e^{ - \alpha t} \left( {1 + \cos \left( {\beta t} \right)} \right)$$

$$y_{212} \left( t \right) =\left( 2r-1 \right) e^{ - \alpha t} \left( {1- \cos \left( {\beta t} \right)} \right)$$

Obviously, $y_{211} \left( t \right) \ge 0$ and $y_{212} \left( t \right) \ge 0$ for $\forall t \ge 0$. Therefore, impulse response of $G_{21} \left( s \right)$ is nonnegative. Impulse response of $G_{22} \left( s \right)$ is also nonnegative since it is a convolution of two nonnegative impulses of $\frac{{\left( {\alpha - p} \right)}}{{\left( {s + p} \right)}}$ and $G_{21} \left( s \right)$. Finally, $G_{2} \left( s \right)$ has a nonovershooting step response, because it has a nonnegative impulse response which is a sum of two nonnegative impulse responses according to Eq. (15). $\square$

### Remarks 2

Noted that no constraints are added to parameter $\beta$ for transfer function $G\left( s \right)$, therefore, it can be specified as a floating parameter in control system design.

Propositions 1 and 2 should be strictly followed for all nonovershooting systems, and Proposition 3 can be applied to nonovershooting control of third order systems. These three propositions lay a foundation for designing of nonovershooting systems by simple controllers.

## Dominant pole control structure

Dominant pole control structure has a natural advantage for realizing nonovershooting control in that: (1) the dominance of the real pole for nonovershooting control can be ensured; (2) dominant pole placement can be realized by simple controllers, such as PID controllers.

**Figure 3:**
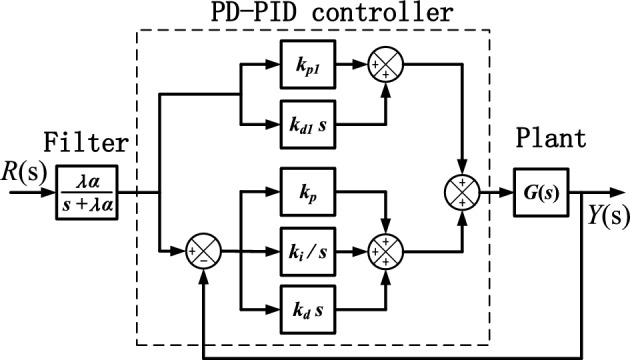
Dominant pole control structure.

To ensure dominance of the dominant poles, the ratio between the real parts of the nondominant poles and that of the dominant poles, denoted as $m$, should satisfy that $m \in \left[ {3, 5} \right]$ or even $m \in \left[ {3, 10} \right]$ ^13,24,25^. Another fact is that complex conjugate poles are easy to be brought in by closed-loop control. In view of above problems, three poles, i.e., one real pole $- \lambda \alpha$ and a pair of complex conjugate poles $- \alpha \pm j\beta$, are adopted as dominant poles for nonovershooting control with $\lambda < 1$ used to increase the robustness of nonovershooting control. The simple controllers used are mainly PD-PID controller, discussed in Du et al.^26^ for its better performance in dominant pole placement, and first order filter. The overall control structure is shown in Fig. 3.

The advantage of control structure in Fig. 3 is that three dominant poles can be designed independently. Dominant real pole $- \lambda \alpha$ can be offered by first order filter while dominant complex poles $- \alpha \pm j\beta$ can be designed in PD-PID control loop. Transfer function of the nonovershooting dominant pole control structure is as follows,

18 $$\frac{Y\left( s \right)}{{R\left( s \right)}} = \frac{{\lambda \alpha \left( {\left( {k_{d} + k_{d1} } \right)s^{2} + \left( {k_{p} + k_{p1} } \right)s + k_{i} } \right)N\left( s \right)e^{ - \tau s} }}{{\left( {s + \lambda \alpha } \right)\left( {sD\left( s \right) + \left( {k_{d} s^{2} + k_{p} s + k_{i} } \right)N\left( s \right)e^{ - \tau s} } \right)}}$$

where $G\left( s \right) = N\left( s \right)e^{ - \tau s} /D\left( s \right)$, and $\tau$ is a time delay.

The zeros brought in by PD-PID controller and the closed-loop poles can be specified respectively. Through placement of the zeros to the nondominant poles that are close to the dominant complex poles, system with a faster response can be designed as the dominant complex conjugate poles can be placed even further from the virtual axis.

*Note *Dominant pole control structure in Fig. 3 can be further simplified without using first order filter, since all three dominant poles can be offered by PD-PID control loop.

## Dominant pole placement

Introduced in this *section* is dominant pole placement method, which can place two poles to the specified points $- \alpha \pm j\beta$ and make other nondominant poles far away from these two poles.

Characteristic equation of PD-PID loop is as follows,

19 $$F\left( s \right) = sD\left( s \right) + \left( {k_{d} s^{2} + k_{p} s + k_{i} } \right)N\left( s \right)e^{ - \tau s} = 0$$

As $- \alpha \pm j\beta$ are two known roots of Eq. (19), when substituted into Eq. (19) two equations can be found. Suppose that $k_{p}$ is a known variable, then $k_{d}$ and $k_{i}$ can be expressed by $k_{p}$, $\alpha$ and $\beta$ as follows:

20 $$k_{i} = \frac{{\alpha^{2} + \beta^{2} }}{2\alpha }k_{p} - \left( {\alpha^{2} + \beta^{2} } \right)A$$

21 $$k_{d} = \frac{1}{2\alpha }k_{p} + B$$

where

$$A = \frac{1}{2\alpha }{\text{Re}}\left( {\frac{ - 1}{{G\left( { - \alpha + \beta j} \right)}}} \right) + \frac{1}{2\beta }{\text{Im}}\left( {\frac{ - 1}{{G\left( { - \alpha + \beta j} \right)}}} \right)$$

$$B = \frac{ - 1}{{2\alpha }}{\text{Re}}\left( {\frac{ - 1}{{G\left( { - \alpha + \beta j} \right)}}} \right) + \frac{1}{2\beta }{\text{Im}}\left( {\frac{ - 1}{{G\left( { - \alpha + \beta j} \right)}}} \right)$$

${\text{Re}}\left( \cdot \right)$ and ${\text{Im}}\left( \cdot \right)$ are functions of finding the real and imaginary part of a complex number correspondingly.

### Dominance checking

For a given $k_{p}$ value, the common practice is to calculate out all the other nondominant poles to check whether their real parts are 3–5 times that of the dominant poles. However, this would be impossible when time delay is included in the closed-loop control, since there are infinite number of poles.

To avoid solving the nondominant poles, Cauchy's argument principle is introduced as follows,

#### Cauchy's Argument Principle

Let Γ be a simply connected region in complex plane ℂ, f : Γ → ℂ analytic in Γ, and $\gamma_{s}$ a simple closed curve in Γ with a counterclockwise direction that does not pass through any zero of f. Let N denotes the total number of zeros of f that located inside of $\gamma_{s}$, then

22 $$N = \frac{1}{2\pi i}\mathop {\oint }\limits_{{\gamma_{s} }}^{{}} \frac{{f^{\prime}\left( s \right)}}{f\left( s \right)}ds$$

Proof of this principle is omitted, interested readers can refer to Kravanja and Van^27^, Vyhlidal and Zitek^28^, and Pekař et al.^29^.

**Figure 4:**
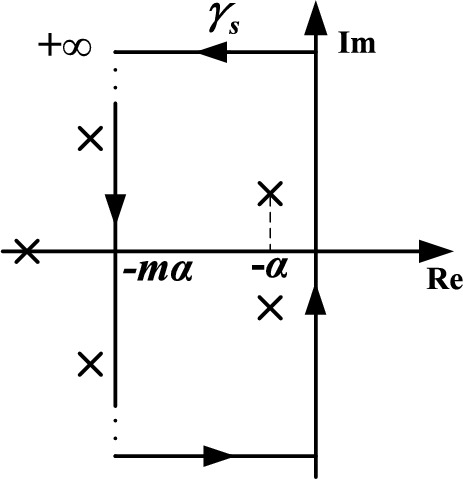
Closed integral curve $\gamma_{s}$ in *s* plane.

By drawing such a closed curve $\gamma_{s}$ in *s* plane in Fig. 4 with $m \ge 3$, only dominant zeros of $F\left( s \right)$ in Eq. (19) will be enclosed if dominant pole placement is fulfilled. Then $N = 2$ can be found after an integration of Eq. (22) along $\gamma_{s}$. Otherwise, $k_{p}$ has to be tuned till the dominance requirements are fulfilled.

### Calculating nondominant poles

To design a system with faster response, two more zeros of $F\left( s \right)$ may be allowed to exist inside $\gamma_{s}$, whose influence can be cancelled by the adjustable zeros introduced by PD-PID. These two zeros, denoted as $- x_{1}$ and $- x_{2}$, can be real or a pair of complex conjugate zeros. Finding these two zeros will be hard when time delay exists in the closed-loop. Thus, a method for calculating $- x_{1}$ and $- x_{2}$, is offered in the following content.

First, draw another closed curve $\gamma_{w}$ that should enclose $- x_{1}$ and $- x_{2}$ with finite size. According to Cauchy–Goursat theorem, the following equation holds,

23 $$\mathop {\oint }\limits_{{\gamma_{w} }}^{{}} \frac{{\left( {s + x_{1} } \right)\left( {s + x_{2} } \right)\left( {s^{2} + 2\alpha s + \alpha^{2} + \beta^{2} } \right)}}{F\left( s \right)}ds = 0$$

Denote $X_{1} = x_{1} + x_{2}$ and $X_{2} = x_{1} x_{2}$, then Eq. (23) can be rewritten as

24 $$X_{1} A_{11} + X_{2} A_{12} = B_{1}$$

where

$$A_{11} = \mathop {\oint }\limits_{{\gamma_{w} }}^{{}} \frac{{s\left( {s^{2} + 2\alpha s + \alpha^{2} + \beta^{2} } \right)}}{F\left( s \right)}ds$$

$$A_{12} = \mathop {\oint }\limits_{{\gamma_{w} }}^{{}} \frac{{\left( {s^{2} + 2\alpha s + \alpha^{2} + \beta^{2} } \right)}}{F\left( s \right)}ds$$

$$B_{1} = - \mathop {\oint }\limits_{{\gamma_{w} }}^{{}} \frac{{s^{2} \left( {s^{2} + 2\alpha s + \alpha^{2} + \beta^{2} } \right)}}{F\left( s \right)}ds$$

In fact, $A_{11}$, $A_{12}$ and $B_{1}$ are values that can be calculated by computers.

Arbitrarily choose a point $x_{0}$ inside $\gamma_{w}$, another equation can be found according to Cauchy’s integral formula as

25 $$\begin{gathered} \mathop {\oint }\limits_{{\gamma_{w} }}^{{}} \frac{{s\left( {s + x_{1} } \right)\left( {s + x_{2} } \right)\left( {s^{2} + 2\alpha s + \alpha^{2} + \beta^{2} } \right)}}{{\left( {s - x_{0} } \right)F\left( s \right)}}ds \hfill \\ \hfill \\ = 2\pi j\frac{{x_{0} \left( {x_{0} + x_{1} } \right)\left( {x_{0} + x_{2} } \right)\left( {x_{0}^{2} + 2\alpha x_{0} + \alpha^{2} + \beta^{2} } \right)}}{{F\left( {x_{0} } \right)}} \hfill \\ \end{gathered}$$

After manipulation, Eq. (25) can be rewritten as

26 $$X_{1} A_{21} + X_{2} A_{22} = B_{2}$$

where

$$A_{21} = \mathop {\oint }\limits_{{\gamma_{w} }}^{{}} \frac{{s^{2} \left( {s^{2} + 2\alpha s + \alpha^{2} + \beta^{2} } \right)}}{{\left( {s - x_{0} } \right)F\left( s \right)}}ds$$

$$A_{22} = \mathop {\oint }\limits_{{\gamma_{w} }}^{{}} \frac{{s\left( {s^{2} + 2\alpha s + \alpha^{2} + \beta^{2} } \right)}}{{\left( {s - x_{0} } \right)F\left( s \right)}}ds$$

$$B_{2} = 2\pi j\frac{{x_{0} \left( {x_{0} + x_{1} } \right)\left( {x_{0} + x_{2} } \right)\left( {x_{0}^{2} + 2\alpha x_{0} + \alpha^{2} + \beta^{2} } \right)}}{{F\left( {x_{0} } \right)}} - \mathop {\oint }\limits_{{\gamma_{w} }}^{{}} \frac{{s^{3} \left( {s^{2} + 2\alpha s + \alpha^{2} + \beta^{2} } \right)}}{{\left( {s - x_{0} } \right)F\left( s \right)}}ds$$

The value of $A_{21}$, $A_{22}$ and $B_{2}$ can also be calculated by computers.

Thus, $- x_{1}$ and $- x_{2}$ can be solved after acquiring the value of $X_{1}$ and $X_{2}$ which can be solved as follows,

27 $$\left[ {\begin{array}{*{20}c} {X_{1} } \\ {X_{2} } \\ \end{array} } \right] = \left[ {\begin{array}{*{20}c} {A_{11} } & {A_{12} } \\ {A_{21} } & {A_{22} } \\ \end{array} } \right]^{ - 1} \left[ {\begin{array}{*{20}c} {B_{1} } \\ {B_{2} } \\ \end{array} } \right]$$

Finally, according to pole-zero cancellation, parameters $k_{d1}$ and $k_{p1}$ can be determined by

28 $$k_{p1} = - \frac{{k_{i} \left( {x_{1} + x_{2} } \right)}}{{x_{1} x_{2} }} - k_{p}$$

29 $$k_{d1} = \frac{{k_{i} }}{{x_{1} x_{2} }} - k_{d}$$

## Illustrating examples

To show the feasibility of our method, two examples are given below.

### Example I

Consider the following unstable plant model with time delay,

30 $$G_{p1} = \frac{1}{{\left( {s^{2} + s + 9} \right)\left( {s - 0.1} \right)}}e^{ - 0.2s}$$

**Figure 5:**
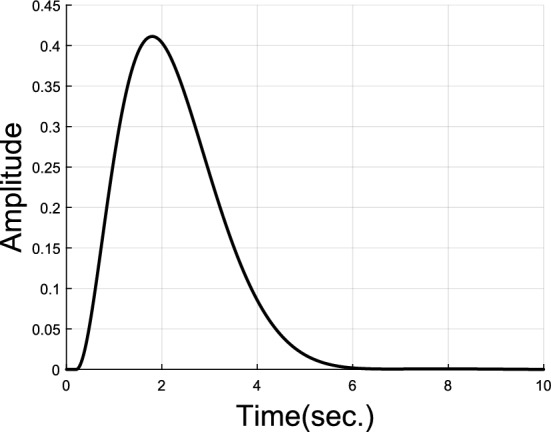
Impulse response of plant model $G_{p1}$.

**Figure 6:**
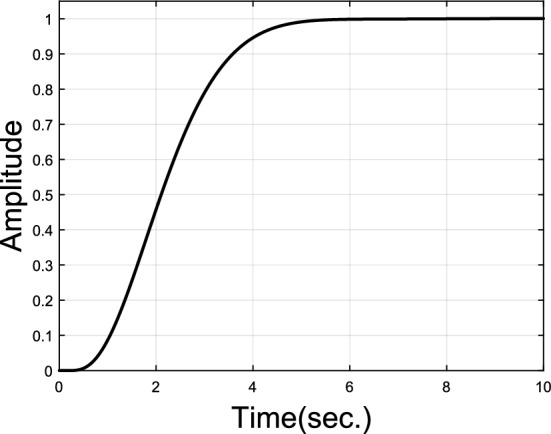
Unit step response of plant model $G_{p1}$.

Nonovershooting control of such complex plant model, especially with time delay, is hard for simple PID controllers. Suppose dominant poles of the inner PD-PID control loop are set to be $- 1.0000 \pm 1.000j$, let $m = 3$, and 2 more poles are allowed to exist inside closed curve $\gamma_{s}$. To satisfy dominant pole placement, $k_{p} \in \left( {1.71,{ }5.52} \right)$ can be found. If $k_{p} = 2$ is chosen, then $k_{i} = 0.2942$ and $k_{d} = - 5.8861$ can be obtained by Eq. (20) and Eq. (21). Two unknown poles close to $- 1.0000 \pm 1.000j$ can be determined to be $x_{1,2} = - 0.2165 \pm 0.3527j$ by method given in Subsection “Calculating nondominant poles”  then $k_{p1} = - 1.2562$ and $k_{d1} = 7.6042$ can be calculated by Eqs. (28) and (29). Parameter $\lambda$ of the first order filter is set to be 0.98. Impulse and step responses of this nonovershooting control system are shown in Figs. 5 and 6.

### Example II

Consider the following 6th order plant model excerpt from Astrom and Hagglund^15^ with a reduction in order.

31 $$G_{p2} = \frac{1}{{\left( {s + 1} \right)^{6} }}$$

**Figure 7:**
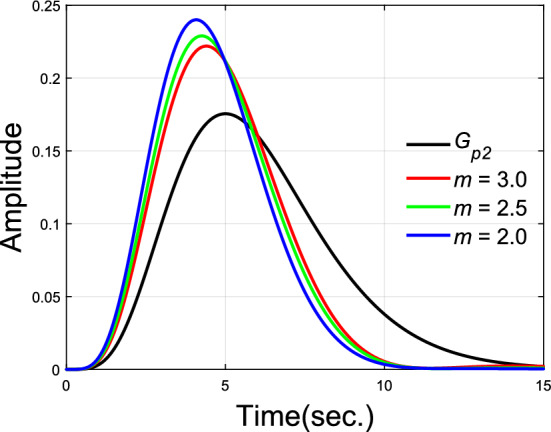
Impulse responses of plant model $G_{p2}$.

**Figure 8:**
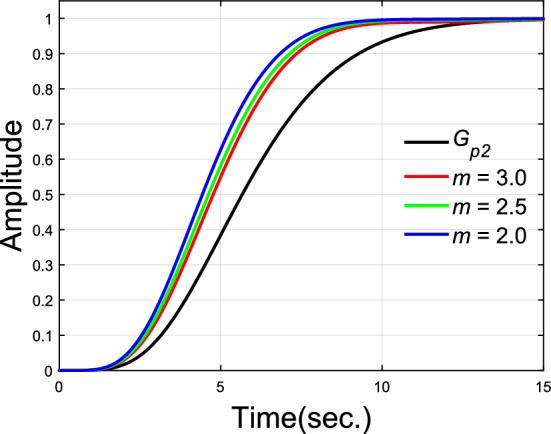
Unit step responses of plant model $G_{p2}$.

As a 6th order transfer function, G_p2_ is already a high order model. And the order of closed-transfer function is up to 7 together with PD-PID controller. Stabilization of such high order systems by simple PID controller is already not easy, let alone nonovershooting control. Due to the restriction of the plant’s high order, placement of the designated dominant pair of poles may fail when larger dominance ratio $m$ is used. Therefore, small $m$ values are checked in this example. The parameters of PD-PID controllers under different $m$ values are given in Table 1. Impulse and step responses of these nonovershooting control systems are shown in Figs. 7 and 8.

**Table 1:** Parameters of PD-PID controllers.

Dominance ratio $m$	Dominant poles	$k_{p}$	$k_{d}$	$k_{i}$	$k_{p1}$	$k_{d1}$	$\lambda$
$3.0$	$0.4750 \pm 0.6500j$	$0.9$	$1.3707$	$0.6154$	$- 0.7603$	$2.9272$	$0.99$
$2.5$	$0.5600 \pm 0.6500j$	$0.7$	$0.8897$	$0.5448$	$- 0.6359$	$3.3829$	$0.99$
$2.0$	$0.6900 \pm 0.7000j$	$0.2$	$0.1760$	$0.3360$	$- 0.0541$	$4.0196$	$0.99$

Compared with the rising time (90%) $9.2747$ s of $G_{p2}$, results show that rising time are improved to $7.3567$ s, $7.1353$ s and $6.8435$ s after control with dominance ratio $m = 3.0$, $2.5$ and $2.0$ separately. Although dominance of the designated dominant poles has been reduced, a good nonovershooting effect can still be maintained in the step responses.

## Conclusions

In this paper, design of simple nonovershooting controllers for linear high order systems with or without time delay are discussed. Three propositions proved in “Basic principles for nonovershooting” Section laid a foundation for realizing nonovershooting control. Under direction of these propositions, dominant pole placement method with modified PD-PID controller and first order filter are devised. Illustrative examples show the effectiveness of our method.

Since PID controllers and first order filters are used, the nonovershooting control method discussed should be more feasible in application compared with other methods leading to complex controllers designed. In addition, the nonovershooting dominant pole control idea can also be used by other controllers, not just limited to PID controllers.

## Data Availability

The authors declare that the data supporting the findings of this study are available within the paper.
